# Co-infections of malaria and dengue in Timika, a highly endemic malaria area in Central Papua, Indonesia

**DOI:** 10.1371/journal.pone.0345164

**Published:** 2026-03-25

**Authors:** Bunga Rana, Leily Trianty, Marsha S. Santoso, Agatha M. Puspitasari, Fahira A. Nisa, Ristya Amalia, Lise Carlier, Kevin Tetteh, Rintis Noviyanti, R. Tedjo Sasmono

**Affiliations:** 1 Exeins Health Initiative, Jakarta, Indonesia; 2 Eijkman Research Center for Molecular Biology, National Research and Innovation Agency, Cibinong, West Java, Indonesia; 3 Foundation for Innovative New Diagnostics (FIND), Geneva, Switzerland; Universitas Padjadjaran, INDONESIA

## Abstract

**Objective:**

Malaria and dengue are two important infectious diseases in Indonesia, but information about their co-infections is still limited. This study aimed to describe malaria–dengue co-infections in Timika, Papua, including the clinical manifestations and genetic characteristics of the infecting dengue virus (DENV).

**Methods:**

Sixty-nine microscopy-confirmed malaria patient samples from Timika, a malaria-highly endemic region in Central Papua province, Indonesia collected in 2022 were screened for DENV infection using qRT-PCR. Malaria speciation was performed using qRT-PCR. The whole genomes of the viruses were sequenced using MinION Oxford Nanopore Technology, and the phylogenetic analysis was inferred to determine the origin, genotype, and genetic characteristics of the viruses.

**Results:**

Out of 69 malaria-positive samples, three patients (4.3%) were co-infected by DENV-2 (1 patient) and DENV-4 (2 patients). The infecting parasites were *P. vivax, P. falciparum*, and *P. malariae* as determined using qRT-PCR. The whole genomes of the infecting DENV-4 were successfully sequenced and phylogenetic analysis revealed the genotype of the virus as Genotype II, which were closely related to DENV strains from Makassar city in South Sulawesi province, Indonesia.

**Conclusions:**

This study demonstrates the occurrence of malaria and dengue co-infections in Timika, Central Papua, Indonesia, with a prevalence of 4.3%. The co-infection poses a unique clinical and diagnostics challenges due to overlapping symptoms and potential complications. This finding highlights the diagnostic difficulties, clinical presentations, and future possibilities for such cases. The genomic data of DENV obtained from these cases provide new information about virus circulation in Indonesia and support the importance of combining clinical and molecular approaches for surveillance.

## Introduction

Malaria and dengue are among the most important and widespread mosquito-borne infections that represent major public health problems especially in tropical and sub-tropical countries [1]. Malaria is an acute febrile illness caused by *Plasmodium* parasite infection transmitted by *Anopheline* mosquitoes which is reported to cause an estimated 219 million cases globally resulting in more than 400,000 deaths every year [2]. Among 200 species of *Plasmodium,* only five species are responsible for malaria infection in humans which are *P. falciparum (Pf), P. vivax (Pv), P. malariae (Pm), P. ovale (Po),* and *P. knowlesi (Pk)* [3]. Meanwhile, dengue is the leading cause of arthropod-borne viral disease in the world, caused by dengue virus (DENV) which is transmitted to humans by *Aedes* mosquitoes [4]. More than 3.9 billion people in over 129 countries are at risk of contracting dengue, with an estimated 40,000 deaths every year [5,6]. DENV is categorized into four genetically and antigenically distinct serotypes which are DENV-1, DENV-2, DENV-3, and DENV-4, and each of the serotypes is further grouped into multiple genotypes based on its genetic diversity [7].

Indonesia still routinely faces malaria as a major burden on public health. The country ranks as the second-largest contributor to malaria cases in Southeast Asia, with an estimated 800,000 malaria cases detected in 2021 [8]. The disease is mostly concentrated in the eastern part of the country, mainly in provinces on Papua Island. In 2023, there has been a significant increase in malaria case examinations in Indonesia, reaching 3,464,738 cases (an increase compared to the previous year) with 418,546 positive cases [9]. Three provinces in Papua Island namely Papua, Central Papua, and South Papua, reported very high Annual Parasite Incidence (API) of 156.59; 103.40; and 82.07 per 1000 population, respectively. These high API in Papua provinces, which are much higher than the other provinces of Indonesia, illustrate the significant contribution to API at the national level [9].

Although Papua is known as a high endemic area for malaria and contributes greatly to malaria cases in Indonesia, this area conversely has the lowest prevalence of dengue, even since dengue was first discovered in Indonesia in 1968 [10–15]. Only since 2023, dengue cases have started to increase in the area, during which West Papua province ranked first in dengue incidence rates in Indonesia [9].

The low endemicity of dengue in Papua in the last decades causes this disease to be neglected. Almost all cases of fever in Papua are usually associated with malaria infection, so that dengue diagnosis is not a priority for local health services. Thus, the possibility of co-infection cannot be ruled out. In spite of co-infection cases of malaria and dengue being reported globally, the incidence remains quite rare, [16,17]^,^ especially in high endemic areas of malaria. The similarity in the clinical manifestations of malaria with dengue (such as fever and headache) often results in misdiagnosis and contributes to disease severity and increased mortality.

This study reports the occurrence of malaria and dengue co-infection in Timika, Mimika Regency, Central Papua Province, Indonesia. The co-infection cases reported in this paper are the second report of malaria and dengue concurrent infection in Papua, as well as the first DENV whole genome sequences isolated from Timika, Papua, Indonesia.

## Materials and methods

### Ethics statement, study design and patients samples

This study is a retrospective observational study on archived samples obtained from a cohort of malaria patients recruited during December 2022 – April 2023 in Timika primary health centre, Mimika Regency, Central Papua province (previously known as Papua province), Indonesia. Study protocol for the examination of archived, unlinked, de-identified patient blood samples for pathogen detection was reviewed and approved by the Health Research Ethics Committee of the National Research and Innovation Agency, Indonesia with approval number 121/KE.03/SK/11/2023.

Demographic, medical history, and symptoms of the patients were recorded. Samples were anonymized and unlinked so there is no information that could identify individual participants during or after data collection. Dengue detection was performed on 69 malaria microscopy-positive samples.

### Malaria diagnosis

Collected patients’ blood were screened for malaria using Bioline™ Malaria Ag *P.f/P.v*. RDT (Abbott, USA). The blood smears of patients were observed under a microscope to detect the *Plasmodium spp*. The parasites’ DNA from patients’ whole blood were extracted using QIAamp DNA Blood Kit (Qiagen, USA) and used in malaria quantitative Real-Time*-*PCR (qRT-PCR) assay to detect and distinguish four *Plasmodium spp*, using method as described previously [18]. Collected blood and plasma samples were stored in – 80ºC until further use.

### Dengue diagnosis

The plasma samples of patients were tested for dengue using Standard F Dengue FIA for NS1 antigen and IgG and IgM antibodies (SD Biosensor, Korea). The results were confirmed with Ultra Dengue IgM/IgG ELISA (SD-Biosensor, Korea). To detect dengue infection, the viral RNA of the samples was extracted using QIAamp Viral RNA Mini Kit (Qiagen, USA). Dengue molecular detection and serotyping was performed using Standard™ M-10 DENV 1–4 RT-PCR (SD Biosensor, Korea) and further confirmed using CDC DENV-1-4 Real Time RT-PCR (Centers for Disease Control and Prevention, USA), performed according to kit’s instruction.

### DENV whole genome sequencing and phylogenetic analysis

DENV whole genome sequencing using MinION (Oxford Nanopore Technology-ONT, UK) was conducted following previously described methods [19]. Reverse transcription was performed on RNA extracted from plasma samples using SuperScript™ III Reverse Transcriptase (Invitrogen-Thermo Scientific, USA). Amplification of the sequence was performed using multiplex PCR using Q5® High-Fidelity DNA Polymerase (New England Biolabs, UK). The quality of the amplicons was evaluated by agarose gel electrophoresis and DNA quantification using Qubit High Sensitivity dsDNA (Invitrogen-Thermo Scientific, USA). The sequencing library was prepared using Native Barcoding Kit 96 V14 (ONT, UK) and sequenced using MinION Mk1B with Flow Cell R10.4.1 (ONT, UK).

The Pod5 data generated through MinKNOW software v23.07.5 (ONT, UK) were base-called using Guppy v6.5.7 (ONT, UK) with super accurate model. Data QC and sequence assembly were performed using BBDuk v38.84 and Minimap2 on Geneious Prime v2023.2.1. The reads were mapped to DENV-4 reference sequence NC_002640. Whole genome sequences of TMK-22-022 and TMK-22-044 were obtained and submitted to GenBank repository and granted accession numbers PQ394951 and PQ394952. The full length of envelope gene (E) of TMK-22-022 and TMK-22-044 were grouped with the reference sequences data set that were downloaded from GenBank and a molecular clock phylogenetic analysis was generated using the Bayesian Markov Chain Monte Carlo (MCMC) algorithm following previous study [20].

## Results

### Dengue detection in malaria-confirmed samples

We conducted dengue qRT-PCR detection in malaria-confirmed archived samples as part of a dengue diagnostics evaluation study. Initially, a total of 69 malaria-confirmed plasma samples were used as non-dengue reference standards to assess the specificity of the evaluated dengue diagnostics. However, we detected three samples (4.3%) were tested positive using Standard™ M-10 DENV 1-4 RT-PCR (SD Biosensor, Korea). The positivity was further confirmed using CDC DENV-1-4 Real Time RT-PCR (US-CDC, USA).

To further investigate the dengue infection, the plasma samples were tested for dengue NS1 antigen and IgM/IgG antibodies using fluorescent-based RDT, however, the detection was negative for all, except for TMK-22-024 which was tested positive for IgG. The IgG and IgM results were reconfirmed using ELISA. Serotyping using qRT-PCR resulting DENV-4 positive for TMK-22-024 and TMK-22-044, and DENV-2 positive for TMK-22-029 (Table 1). Altogether, the results demonstrate the occurrence of dengue co-infection in malaria patients attending primary health centre in Timika, Central Papua.

**Table 1:** Summary of co-infection cases characteristics in this study.

No	Patient ID	Age	Sex	Malaria Detection			Dengue Detection				
				RDT	Microscopy	qRT-PCR	NS1	IgM	IgG	Serotype	Genotype
1	TMK-22–024	7	M	–	*Pv*	*Pv*	–	–	–	DENV-4	II
2	TMK-22–029	46	F	–	*Pf*	*Pv*	–	–	+	DENV-2	N/A
3	TMK-22–044	25	M	–	*Pm*	*Pf, Pm, Pv*	–	–	–	DENV-4	II

*Pv: P. vivax; Pf: P. falciparum; Pm: P. malariae.*

### Diagnostic and clinical characteristics of co-infection patients

**Fig 1:**
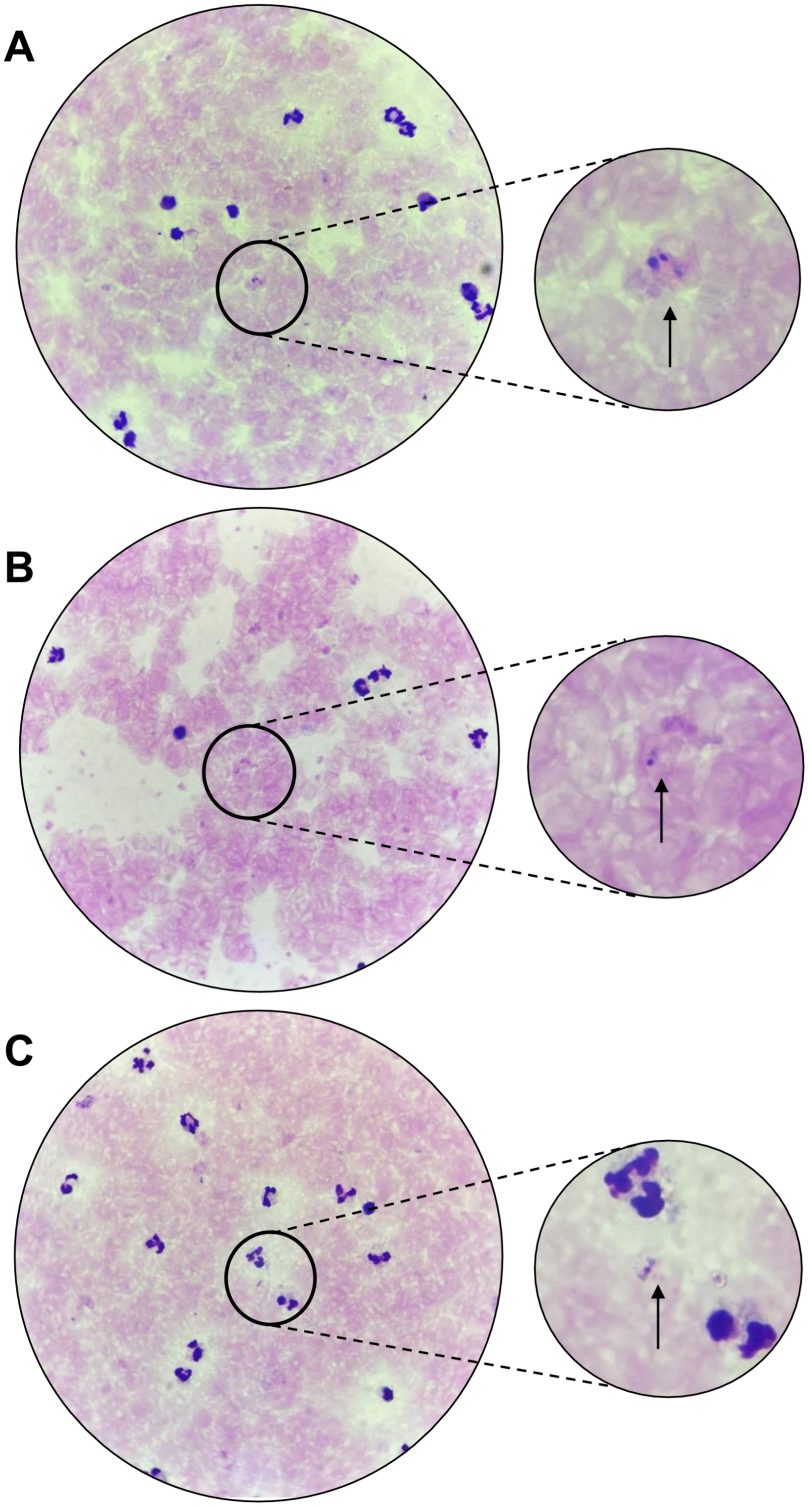
Microscopy detection of *Plasmodium spp* in blood smears of patients. **A.** TMK-22-024; **B.** TMK-22-029; **C.** TMK-22-044.

To fully understand the co-infection characteristics, we retrieved the diagnosis and clinical data of the patients. The first patient (TMK-22-024) was a seven-year-old boy who came to Timika Primary Health Center on 23^rd^ December 2022 presented with a fever and headache starting two days prior to admission. Microscopy examination of blood smear confirmed *P. vivax* infection in this patient (Fig 1A) with a parasitemia level of 136 parasites/µL. qRT-PCR was performed and confirmed the presence of *P. vivax* with a Ct value of 26.93, supporting the microscopy findings.

The second patient (TMK-22-029) was a 46-year-old female reported dizziness, nausea, fatigue, headache, and vomiting, with fever beginning at least two days prior to her blood draw on 23^rd^ December 2022. The patient had malaria infection history in the past 6 months. Initial microscopy of this patient detected a *P. falciparum* infection with a parasitemia level of 24 parasites/µL (Fig 1B). However, qRT-PCR assay identified *P. vivax* as the causative agent, suggesting possible mixed or sequential infection with *P. falciparum* and *P. vivax.*

The third patient (TMK-22-044) was a 25-year-old male who came to the same primary health centre on 27^th^ December 2022 presented with high-grade fever, abdominal pain, sweats, dizziness, nausea, stomach-ache, fatigue, and headache. This patient had malaria infection history in the past 3 years. Microscopy examination detected *P. malariae* infection with an average of 112 parasites/µL (Fig 1C). Confirmation using qRT-PCR resulting the sample as positive for *P. vivax* with mix infection of *P. falciparum* and *P. malariae.* The diagnosis summary is presented in Table 1.

### DENV genome sequencing and phylogenetic analysis

**Fig 2:**
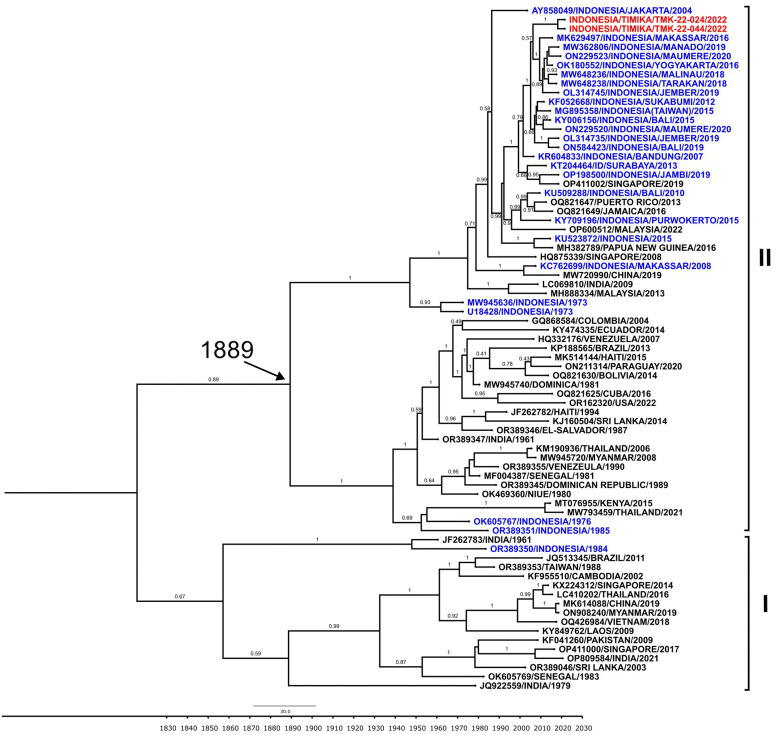
Phylogenetic analysis based on Envelope gene (E) sequences of DENV-4 from Timika (red) showing the evolutionary relationship with strains from other cities in Indonesia (blue) and other countries (black), grouped into genotypes shown in Roman numerals.

To further confirm the dengue and malaria co-infection, we conducted DENV whole genome sequencing of the samples. Of three samples, only two were able to be fully sequenced, i.e., DENV-4 from TMK-22-024 (10,195 nt) and TMK-22-044 (10,530 nt). The DENV-2 from TMK-22-029 was not successfully sequenced. Phylogenetic analysis was then performed to determine the genotype and geographical origin of the Timika DENV-4. As shown in Fig 2, both of DENV-4 isolates from Timika belonged to Genotype II and shared 99.9% similarity of each other. These isolates formed a monophyletic group together with isolates from Indonesian cities of Makassar (2016) [21], Manado (2020) [22], and Maumere (isolated in 2020) and close to isolate from Sukabumi [23] isolated in 2019 with 99.2% similarity.

## Discussion

Malaria and dengue are two febrile mosquito-borne diseases that contribute a high public health burden in tropical regions. While classically the malaria parasite is transmitted by the ‘jungle mosquito’ *Anopheles* species and dengue virus is transmitted by the ‘urban mosquito’ *Aedes aegypti*, both diseases co-circulate and are endemic especially in areas near to jungles that are undergoing urbanization [1]. The shift in jungle and urban area may influence the mosquito habitat in Mimika regency, making it more feasible for dengue vectors to live while it is still a large habitat of malaria vectors with the circulating parasites. Altogether, this result might indicate the possibility of double burden infection of malaria and dengue in Papua, as well as the higher chance of co-infections.

Up to now, we found only six malaria and dengue co-infection case reports in Indonesia, including those from Makassar, [24] Surabaya, [25] and Aceh [26]. Most reported cases of malaria and dengue co-infection in Indonesia and neighbouring Asian countries involved patients with travel histories to or from malaria-endemic areas (S1 Table). From previous studies in Asia, Africa, and South America, rates of malaria and dengue co-infection is about less than 10% of enrolled febrile cases [27]. Our findings showed a malaria-dengue co-infection rate of 4% (3/69), though it is a limitation of our study that the number of samples included are relatively small and that it was not initially designed to be an incidence study. An accurate incidence rate of malaria-dengue co-infection is difficult to ascertain as it is uncommon for confirmatory diagnostic detection of malaria and dengue to be performed simultaneously, especially in limited clinical settings. Once a patient is diagnosed with either malaria or dengue, further observations of concurrent infections are not commonly explored unless severe and/or unusual complications develop (S1 Table). Furthermore, malaria and dengue share similar clinical presentations [28,29], making it difficult to distinguish cases of co-infection from those of malaria or dengue mono-infection.

In our study, the diagnosis of dengue in addition to malaria was discovered after the fact; at the time of admission to the primary health centre, the patients were only diagnosed as having malaria. All three patients had fever and non-specific symptoms that could suggest either dengue or malaria but being in an area highly endemic to malaria and with low endemicity for dengue, the first etiology explored by clinicians was malaria. According to the WHO guideline, in malaria-endemic areas, malaria should be suspected in any patient presenting with a history of fever with no other obvious case [30].

Several studies including a systematic review and meta-analysis study revealed that patients with malaria and dengue co-infection can develop either severe malaria or severe dengue, with a prevalence estimate of severe malaria among co-infection patients was 32% [31]. The study also revealed that patients with malaria and dengue co-infection had a higher risk of severe dengue than those with dengue mono-infection. In our study, case TMK-22-029 reported vomiting and case TMK-22-044 reported having abdominal pain, which are dengue warning signs that may need continuous observation and medical intervention if the symptoms are severe and persistent [32]. Cases TMK-22-024 and TMK-22-044 had negative dengue IgG results and were retrospectively discovered to have primary dengue infection, which are usually milder in symptoms or asymptomatic.

The treatments for malaria and dengue are very different; malaria requires chemotherapy specific to the *Plasmodium* species, while dengue treatment is supportive according to clinical manifestations [30,32]. Accurate diagnosis is important to prevent mistreatment, prolonged hospitalization, and development of complications. Missed malaria diagnosis, particularly *Plasmodium falciparum*, can lead to cerebral malaria, while missed dengue diagnosis can lead to severe capillary leakage, both of which can be fatal. All three patients in our study were discharged as outpatient cases and given oral treatment as per protocol for their respective *Plasmodium* species. There were no follow-ups on patient outcome, though the prognosis of recovery appeared to have been good based on the discharge diagnosis. Our findings suggest an alert for health care professionals and the government for the possibilities of malaria and dengue co-infections in malaria-endemic areas, especially when the dengue cases increased.

Our study detected DENV-2 and DENV-4 circulating concurrently with the Plasmodium species already known to circulate in Timika such as *P. falciparum, P. vivax, P. malariae,* and *P. ovale* [33]. Case TMK-22-044 of our study had mixed malaria infection of *P. falciparum, P. malariae,* and *P. vivax* according to qRT-PCR result, in addition to DENV-4. Mixed *Plasmodium* spp. infections can frequently occur in endemic areas of malaria [34]. Being the only region in Indonesia on which all the cities/districts have not achieved malaria elimination, [14] and contributing for almost 90% malaria cases in Indonesia, [2] mix-infection in Papua region is expected to be found. As only low proportion of infection is detected by microscopy, which might be due to low parasite densities, observer error, and technical difficulties, mix-infection are frequently unrecognized or underestimated [34–36].

To further understand the genetic characteristics of DENV-4 involved in co-infection, we sequenced the whole genomes of the viruses and successfully obtained two DENV-4 whole genome sequences from TMK-22-024 and TMK-22-044. These sequences are the first DENV-4 genome sequences originated from Timika, providing important genetics information of the viruses circulating in the region. All two Timika isolates belonged to DENV-4 Genotype II, which is the most common circulating DENV-4 genotype in Indonesia [37–39]. Based on phylogenetic analysis (Fig 2), the Timika DENV-4 isolates are closely related to strains from Indonesian cities of Makassar, Manado, and Maumere. Makassar and Manado are among the largest cities in eastern Indonesia and relatively close to Timika although in different island. Intensive people migration among the cities most likely contribute to the spread of DENV to Timika. In terms of genome similarity, these two isolates are highly similar. They were isolated from patients attended one health center in the different time, which indicates that the viruses are locally transmitted and have been circulating in Timika for a while.

## Conclusions

This study detected three cases of malaria and dengue co-infection in Timika, Central Papua, confirming the concurrent dengue and malaria transmissions in the region. The finding highlights the need to strengthen differential diagnosis strategies in malaria-endemic setting with changing arboviral transmission patterns. The overlapping clinical symptoms between dengue and malaria underscore the potential misdiagnosis and inappropriate clinical management. The health authorities should consider implementing integrated disease surveillance, vector control, and adopt multi-disease diagnostic approaches to ensure accurate diagnosis and optimal patient management in malaria-endemic area in Indonesia.

## Supporting information

S1 Table Malaria and dengue co-infection reported cases. Summary of malaria and dengue co-infection in Indonesia and neighboring Asian countries.
(PDF)
