# Supplementary material for: Co-infections of malaria and dengue in Timika, a highly endemic malaria area in Central Papua, Indonesia
Source: PLoS One. 2026 Mar 25;21(3):e0345164. doi: 10.1371/journal.pone.0345164 (PMC13016352; doi:10.1371/journal.pone.0345164)
Supplement: S1 Table — Summary of malaria and dengue co-infection in Indonesia and neighboring Asian countries. (PDF) [file pone.0345164.s001.pdf]

S1 Table. Malaria and Dengue co-infection reported cases in Indonesia and neighboring Asian countries.

No	Reference	Sex	Age	Mobility		Clinical Symptoms	Dengue Detection		Malaria Detection		Diagnosis	Notes	Outcome
				**Location**	**Travel History/origin**		**NS1**	**IgG/IgM**	**RDT**	**Microscopy**			
1	Thaha *et al*. 2008	N/A	N/A	Indonesia	not described	Fever	N/A	IgG Positive	N/A	*P. falciparum*	DSS and *P. falciparum* malaria; acute renal failure	The patient had a history of repeated malaria infection	Discharged after examinations showed positive progress
2	Santoso *et al.* 2024	Male	26	Jombang, Indonesia	Kalimantan, Indonesia	Chills and stiffness, a positive result of the rumple leed test.	N/A	IgG and IgM positive	Positive *P. falciparum*	*P. falciparum*	DSS and *P. falciparum* malaria		N/A
3	Pelupessy *et al*. 2023	Male	14	Makassar, Indonesia	Papua, Indonesia	High fever and chills, severe headache, photophobia, abdominal tenderness, positive result of the rumple leed test	N/A	IgG positive	N/A	*P. falciparum*	Dengue with warning signs and *P. falciparum* malaria	Having a longer fever after dengue infection treatment, clinical improvement was observed after receiving malaria medication.	Discharged after 5 days of treatment
4	Viryani et al. 2024	Male	42	Surabaya, Indonesia	Papua, Indonesia	Fever, fatigue, pain at the joints, headache, nausea, and loss of appetite. Nosebleed, coughing fresh blood, red spot on the leg, tightness, yellowish eyes, brownish urine and stool	N/A	IgG and IgM positive	N/A	*P. vivax, P. ovale*	Mixed type severe malaria co-infection with dengue and has complications of expanded dengue syndrome	Severity symptoms of dengue infection. The patient was an Indonesia National Armed Forces in Malaria-endemic region (Papua).	Discharged after 14 days of treatment
5	Khairi *et al.* 2024	Male	36	Banda Aceh, Indonesia	Mountainous area of Lhoknga, Indonesia	Fever, hyperemic conjunctiva, pain throughout the body, vomiting, chills, sweating	Positve	IgG positive	N/A	*P. knowlesi*	DHF Grade I and *P. knowlesi* malaria	DHF was detected first, the symptoms didn't reduce until malaria infection was found and anti-malarial drug regimen were given	N/A
6	Weng *et al.* 2021	Male	54	Malaysia	Pakistan (1 year prior to infection)	Fever associated with chills and rigors for three days before admission, three episodes of vomiting, and reduced appetite for a similar period.	Positive	IgM Positive	N/A	*P. vivax*	Dengue and *P. vivax* malaria	Possibility of *P. vivax* relapse	Discharged after treatment
7	Rahim *et al*. 20017	Male	59	Malaysia	Forest in Jeli, Kelantan, Malaysia	High-grade fever with chills and rigors, dry cough, myalgia, arthralgia, chest discomfort and poor appetite of 1 week duration	Positive	N/A	N/A	*P. knowlesi*	Severe dengue and *P. knowlesi* malaria; multi-organ failure	The patient was treated as having severe dengue with plasma leakage complicated with type 1 respiratory failure. The platelet count remained low, although dengue infection was in the recovery phase, and detected to be malaria positive	Discharged after 16 days of treatment
8	Chong *et al.* 2017	Male	59	Kota Bharu, Malaysia	Jeli (origin), Malaysia	Fever, headache, myalgia, arthralgia, and poor oral intake over the 6 days before admission. Lethargic, mildly dehydrated, mildly jaundiced, and the abdomen was soft with no organomegaly.	Positive	IgM Positive	N/A	*P. knowlesi*	Severe dengue *and P. knowlesi* malaria; multi-organ failure	Treated for severe dengue with compensated shock, but the patient had unresolved acidosis as well as multi-organ failure involving respiratory, renal, liver, and haematological systems. Further malaria testing confirmed co-infection with malaria *P. Knowlesi*. Highlighting the dangers of delay diagnosis	Discharged after 15 days of treatment
9	Issaranggoon *et al.* 2014	Male	11	Northern Part of Thailand	N/A	Fever and chills, stomach ache, vomit, myalgia,	Positive	IgM Positive	N/A	*P. falciparum*	Dengue and *P. falciparum* malaria		Discharged after 8 days of treatment
10	Yong *et al.* 2012	Male	49	Singapore	Tanjung Pinang, Riau (two weeks before)	Six-day history of fever, chills, and rigors associated with generalized myalgia that was especially painful over the bilateral thighs. Two episodes of non-bilious, non-bloody vomiting three days before admission and mild dyspnea one day earlier.	Negative	IgM Positive	N/A	*P. falciparum*	Malaria *P. falciparum* with dengue co-infection and past infection of murine typhus, acute kidney injury	*P. falciparum* malaria was detected and treated first, but myoglobinuria and acidosis worsened. Further testing confirmed dengue co-infection	Discharged after treatment

References:

1. Thaha M, Pranawa, Yogiantoro M, Tanimoto M, Tomino Y. Acute renal failure in a patient with severe malaria and dengue shock syndrome. *Clin Nephrol*. 2008;70(5):427-430. doi:10.5414/cnp70427

2. Santoso AA, Setyawati DM, Aulia FA, Burhanuddin M. Experience in Laboratory Diagnostics of Falciparum Malaria and Dengue Co-infection with Eosinophilia: Case Report. *J Diverse Med Res Medicosphere*. 2024;1(4):23-30. doi:10.33005/jdiversemedres.v1i4.16

3. Pelupessy NM, Latief LI, Artati RD, Maulani D. Dengue and Malaria Coinfection in An Overweight Child. *J Med Res*. 2023;9(2):23-25. doi:10.31254/jmr.2023.9204

4. Viryani NM, Bramantono, Hadi U. A Rare Case of Mixed Type Severe Malaria Co-infection with Dengue Complicated by Expanded Dengue Syndrome. *Int J Res Publ*. 2021;74(1). doi:10.47119/IJRP100741420211866

5. Khairi AB, Sihite PL, Azalia R. Concurrent Infection of Dengue and Malaria: An Unusual Case Report. *Indones Health J*. 2024;3(1):150-155. doi:10.58344/ihj.v3i1.306

6. Weng EHQ, Wee CS, Jian LQ. An Unusual Case of Concurrent Dengue and Malaria Infection. *Borneo J Med Sci BJMS*. 2021;15(1):57-57. doi:10.51200/bjms.v15i1.1856

7. Rahim, M, Mohammad N, Besari AM, Ghazali WSW. Severe Plasmodium knowlesi with dengue coinfection. *BMJ Case Rep*. Published online February 20, 2017. doi:10.1136/bcr-2016-218480

8. Chong SE, Zaini RHM, Suraiya S, Lee KT, Lim JA. The dangers of accepting a single diagnosis: case report of concurrent Plasmodium knowlesi malaria and dengue infection. *Malar J*. 2017;16(1):2. doi:10.1186/s12936-016-1666-y

9. Issaranggoon na ayuthaya S, Wangjirapan A, Oberdorfer P. An 11-year-old boy with Plasmodium falciparum malaria and dengue co-infection. *BMJ Case Rep*. 2014;2014:bcr2013202998. doi:10.1136/bcr-2013-202998

10. Yong KP, Tan BH, Low CY. Severe falciparum malaria with dengue coinfection complicated by rhabdomyolysis and acute kidney injury: an unusual case with myoglobinemia, myoglobinuria but normal serum creatine kinase. *BMC Infect Dis*. 2012;12:364. doi:10.1186/1471-2334-12-364
